# Identification and validation of Birc5 as a novel activated cell cycle program biomarker associated with infiltration of immunosuppressive myeloid‐derived suppressor cells in hepatocellular carcinoma

**DOI:** 10.1002/cam4.6271

**Published:** 2023-06-16

**Authors:** Yun Liu, Xi Chen, Wenwu Luo, Yufei Zhao, Björn Nashan, Lei Huang, Xiaodong Yuan

**Affiliations:** ^1^ Department of Radiation Oncology, Anhui Provincial Cancer Hospital The First Affiliated Hospital of USTC, Division of Life Sciences and Medicine, University of Science and Technology of China Hefei China; ^2^ Department of Gastrointestinal Oncology Surgery, Anhui Provincial Cancer Hospital The First Affiliated Hospital of USTC, Division of Life Sciences and Medicine, University of Science and Technology of China Hefei China; ^3^ Department of Pathology The First Affiliated Hospital of Anhui Medical University Hefei China; ^4^ Organ Transplant Center, Department of Hepatobiliary and Transplantation Surgery, The First Affiliated Hospital of USTC Division of Life Sciences and Medicine, University of Science and Technology of China Hefei China; ^5^ Department of Oncology Ruijin Hospital, Shanghai Jiao Tong University School of Medicine Shanghai China; ^6^ Medical Center on Aging of Ruijin Hospital, MCARJH, Shanghai Jiaotong University School of Medicine Shanghai China

**Keywords:** Birc5, cell cycle, hepatocellular carcinoma (HCC), immunotherapy, myeloid‐derived suppressor cells (MDSCs), tumor microenvironment

## Abstract

**Background:**

Preclinical studies and clinical trials have demonstrated that tumor‐intrinsic activation of the cell cycle program impedes anticancer immunotherapy. Identification of cell cycle‐related biomarkers may provide novel therapeutic targets to augment the efficacy of immunotherapy in hepatocellular carcinoma (HCC).

**Method and Results:**

Based on the genes related to cell cycle program, two clusters (Cluster 1 and Cluster 2) were detected in HCC patients via non‐negative matrix factorization algorithm. Multivariable‐adjusted Cox regression analysis indicated that the cell cycle gene‐based classification was a significant prognostic factor for predicting the clinical outcome of HCC patients. Cluster 1 showed shorter overall survival time and progression‐free interval time was associated with activated cell cycle program, higher infiltration of myeloid‐derived suppressor cells (MDSCs) and less sensitivity to immunotherapy. A three‐gene prognostic model, including *BIRC5*, *C8G*, and *SPP1*, was constructed to characterize the cell cycle‐based classification of HCC, which had strong robustness and a stable predictive performance. Notably, Birc5 was positively correlated with CD11b expression (a MDSC marker) in HCC tissue. Concordant high expression of Birc5 and intratumor infiltration level of MDSCs were correlated with worse prognosis of HCC patients. In vitro, hepatocellular Birc5 overexpression promoted immunosuppressive CD11b^+^CD33^+^HLA‐DR^−^MDSC expansion from human peripheral blood mononuclear cells. Genetically modified animal model of liver cancer revealed that *Birc5* depletion upregulated the genes related to lymphocyte‐mediated immunity, natural killer cell‐mediated immunity, interferon‐gamma production, T‐cell activation, and T‐cell‐mediated cytotoxicity. These results suggest an immunosuppressive function of Birc5 in HCC.

**Conclusion:**

Birc5 was a potential biomarker and inducer of intratumor infiltration of MDSCs, which led to T cell exclusion or dysfunction in tumor immune microenvironment, consequently resulting in reduced response to ICIs in HCC.

## Graphical abstract

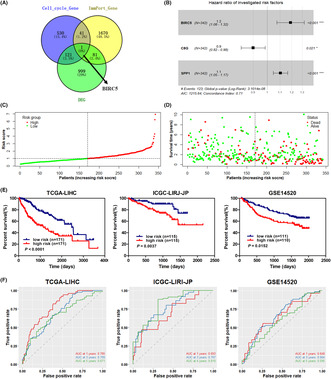

## 1 INTRODUCTION

Hepatocellular carcinoma (HCC) is the most common form of liver cancer, and therapeutic interventions, including surgical resection, loco‐regional percutaneous ablation, and chemotherapy, have limited effects on HCC with a high rate of recurrence within 5 years.^1^, ^2^, ^3^ For patients with HCC who are diagnosed at advanced stages and not suitable for surgery, their median survival time is generally around 6 months.^4^, ^5^ Therefore, the overall survival (OS) of HCC patients is still far from satisfactory. During the last decade, immunotherapy has become a powerful weapon for cancer treatment and revolutionized the systemic management of advanced cancers (e.g., melanoma and renal cell carcinoma).^6^, ^7^ However, the response rate of immune checkpoint inhibitors (ICIs) targeting CTLA‐4 or PD‐1/PD‐L1 in HCC is only 15%–20%, highlighting the urgent need to identify biomarkers that can robustly predict the effectiveness of ICI therapy.^8^ Beyond that, clinical researches have uncovered that the immune cell composition of HCC is markedly associated with survival outcomes and response to immunotherapy.^8^, ^9^, ^10^, ^11^ Hence, it is proposed that biomarker‐directed therapy with good patient stratification and prudent selection of therapeutic agent is important for enhancing the efficacy of immunotherapy against HCC.^6^, ^8^, ^10^

Genes related to cell cycle program are frequently mutated in human neoplasms. Currently, increasing evidence has revealed that genes related to cell cycle program are capable of remodeling tumor microenvironment (TME) and can regulate tumor immune responses, underscoring the potential strategies of targeting cell cycle‐associated genes for immunotherapy of human cancers.^12^, ^13^ For instance, inhibiting the expression of *CDKN2A*, a member of cyclin‐dependent kinase (CDK) inhibitors, could lead to invasion, progression, and immunotherapy resistance in melanoma.^14^, ^15^, ^16^ *CDKN2A* deletion repressed T‐cell infiltration by suppressing chemokine expression in a cell cycle‐dependent manner and might account for immunotherapy failure.^15^ Besides, *TP53*, associated with *STK11* and *EGFR*, has been proved to be a major determinant of tumor immune profile. Previous research showed that patients with *TP53* mutation and without alteration in *STK11* and *EGFR* had the highest levels of CD8^+^ T‐cell and PD‐L1 expression and exhibited best responses to PD‐1 blockade.^17^ In another study, *TP53* mutation was related to activated immune cell infiltration and was regarded as a potential biomarker of immunotherapy in lung adenocarcinoma.^18^ Moreover, retinoblastoma 1 (RB1), the downstream target of CDK4/6, was associated with immunological features of tumor tissues.^19^, ^20^, ^21^, ^22^, ^23^ RB1‐dependent cell cycle inhibition with CDK4/6 inhibitors increased cytotoxic T cell influx and limited intrinsic immunological exclusion in tumors.^19^, ^20^ However, the role of cell cycle‐associated genes in remodeling immune profile and their impacts on immunotherapy efficiency in HCC have been rarely investigated. Hence, further exploiting the impact of cell cycle program on immunological features may provide novel approaches to enhance sensitivity to immunotherapy and develop more durable combinatorial therapies in HCC.

In this study, transcriptome analysis was performed to classify HCC into two clusters based on the genes related to cell cycle program via non‐negative matrix factorization (NMF) algorithm. Next, the associations of the clusters with the prognosis and clinical features of HCC patients were evaluated. Integrated analysis was further conducted to explore the differences in immunogenomic profile and benefits of immunotherapy between the cell cycle gene‐based subtypes. Then, a 3‐gene prognostic model, including *BIRC5*, *C8G*, and *SPP1*, was constructed and validated in different datasets, which could better predict the prognosis of liver cancer. Finally, we identified and validated that Birc5 expression might serve as a prognostic biomarker associated with the infiltration of immune‐suppressive myeloid‐derived suppressor cells (MDSCs) and a promising therapeutic target for HCC patients.

## 2 MATERIALS AND METHODS

### 2.1 Workflow and data collection

The overall workflow of this research is presented in Figure S1. Clinical information and RNA sequencing data of HCC were retrieved from Gene Expression Omnibus (GEO) repository (https://www.ncbi.nlm.nih.gov/geo/), International Cancer Genome Consortium (https://dcc.icgc.org/) and The Cancer Genome Atlas (TCGA) data portal (https://portal.gdc.cancer.gov/), patients from which were named as GSE14520 cohort (*n* = 221), ICGC (LIRI‐JP) cohort (*n* = 231) and TCGA‐LIHC cohort (*n* = 342), respectively. Gene expression profiles of mouse liver cancers with or without *BIRC5* depletion were downloaded from the GEO repository (GSE64804).^24^ The TCGA‐LIHC cohort was employed for data training and internal validation, while the ICGC (LIRI‐JP) and GSE14520 cohorts were used for external validation. 693 reactome cell cycle‐related genes were compiled from MsigDB (https://www.gsea‐msigdb.org/gsea/msigdb/).^25^ The somatic mutation data of patients from the TCGA‐LIHC cohort were downloaded from the TCGA database. The frequencies of genes and mutation types were determined using the maftools package in R.^26^

### 2.2 Consensus molecular subtyping with NMF

NMF was performed to cluster TCGA‐LIHC samples into cell cycle‐related subtypes.^27^ Consensus clustering was conducted using NMF package in R, with a setting of standard “brunet” and 50 iterations. The optimal number of subtypes was determined based on the cophenetic, dispersion, and silhouette coefficients, and the optimal clustering number was selected as 2. Then, the patients in TCGA‐LIHC cohort were categorized into 2 molecular subtypes, termed Cluster 1 (166 patients, 48.5%) and Cluster 2 (176 patients, 51.5%).

### 2.3 Development and validation of a prognostic cell cycle‐related gene signature

1793 immune‐associated genes were downloaded from the ImmPort database (https://www.immport.org/shared/genelists).^28^ Veen diagram revealed that 82 genes were common between these immune‐related genes and the 1202 DEGs. Finally, 24 immune‐related genes with log_2_(count+1) value not less than 1 in all TCGA‐LIHC samples were applied for univariable survival analysis using Cox proportional hazards model and were further integrated into the least absolute shrinkage and selection operator (LASSO) regression analysis. Multivariable survival analysis was conducted to investigate the overall effect of the immune‐related genes on prognosis using the survival package in R software. The prognostic risk score (RS) model was constructed according to the multivariable co‐efficiency multiplied by expression data. The RS model was further evaluated and validated with ICGC (LIRI‐JP) and GSE14520. The median RS of all patients from each dataset was used as a cutoff value to categorize the whole group into low‐ and high‐risk groups. To verify the role of prognostic features, the area under the curve (AUC) was constructed using the timeROC package in R software.

### 2.4 Human specimens

Paired tumor and non‐tumor tissues from 28 HCC patients were recruited from the First Affiliated Hospital of USTC between October 2020 and August 2021 for immunohistochemical analyses. Inclusion criteria for HCC patients were histopathological diagnosis of HCC. The clinical information of the enrolled HCC patients is presented in Table S1. Blood specimens were additionally collected from 6 healthy people without any history of infectious diseases within last year and without any concurrent diseases. The study protocol involving human subjects was approved by the Ethics Committees of the First Affiliated Hospital of USTC (2021‐RE‐113). All participation gave written informed consent in accordance with the Declaration of Helsinki.

### 2.5 Immunohistochemistry analysis

Immunohistochemical staining was conducted according to a previous method.^29^ Five‐millimeter serial sections were prepared from formalin‐fixed and paraffin‐embedded tissues, followed by deparaffinization and rehydration. Antigen retrieval was conducted with sodium citrate buffer (0.01 M, pH 6.0) for 10 min at 98°C. The endogenous peroxidase activity was suppressed by 3% hydrogen peroxide in dH_2_O for 10 min, and then incubated with 5% normal goat serum for 30 min. The serial sections were further incubated with polyclonal rabbit anti‐Birc5 (1:100 dilution; 10508, Proteintech) or anti‐CD11b antibody (1:100 dilution; 21851, Proteintech) at room temperature for 4 h. The sections were exposed to HRP‐conjugated anti‐rabbit secondary antibody for 1 h. After staining with 3,3′‐diaminobenzidine, the sections were counterstained with hematoxylin. Five randomly selected visual fields (×200 magnification) were counted per each section and ImageJ software^30^ was utilized to evaluate the area percentage of positive staining area of Birc5 and CD11b.

### 2.6 Cell culture and transfection

The HCC cell line Huh7 and the immortal human liver cell line THLE‐3 were obtained from the American Type Culture Collection and cultured in high‐glucose DMEM (Invitrogen/Thermo‐Fisher‐Scientific) containing 10% FBS (Gibco). All cells were maintained at 37°C in a humidified incubator with 5% CO_2_. Lentiviral vectors carrying the *BIRC5* gene and control lentiviral vector (Hunan Fenghui Biotechnology Co., Ltd) were added into the THLE‐3 cells. The transfection efficiency was evaluated by western blotting. Fresh peripheral blood mononuclear cells (PBMCs) were cultured in DMEM medium containing 10% FBS (Gibco), 1 mM sodium pyruvate (Gibco) and 2 mM l‐glutamine (Gibco).

### 2.7 Flow cytometry analysis and evaluation of MDSC expansion

PBMCs were isolated from the buffy coats of anonymous human healthy donors using Ficoll‐Paque Premium (GE Healthcare). PBMCs were treated with the supernatant collected from THLE‐3 cells expressing Birc5 or control vector for 5 days. For flow cytometry analysis, cells were collected and washed and incubated with mouse serum (BioLegend) for 10 min at 4°C to block Fcγ receptors. Then, the cells were stained by anti‐human myeloid markers CD11b, CD14, CD33 and HLA‐DR, followed by flow cytometry analysis. Flow cytometry data were acquired using the CytoFLEX (BECKMAN COULTER), and the percentages of proliferating cells were analyzed by FlowJo software 10 (Tree Star, Inc.).

### 2.8 Western blot analysis

Western blotting was conducted according to a previous method.^31^ Total cell protein lysates were extracted on ice using the protease inhibitor cocktail‐containing radioimmunoprecipitation assay buffer. Protein concentrations were detected with a Bio‐Rad protein assay. Then, 10–30 μg of total cell protein extracts were subjected to 10% SDS‐PAGE and electroblotted onto PVDF membranes (Millipore). The membranes were exposed to primary antibodies at 4°C overnight, followed by HRP‐linked anti‐mouse and anti‐rabbit antibodies for 2 h. Primary antibodies used were rabbit anti‐Birc5 polyclonal antibody (10508, Proteintech), mouse anti‐PD‐L1 monoclonal antibody (66248, Proteintech), mouse anti‐GAPDH (60004, Proteintech) and alpha‐Tubulin (66031, Proteintech).

### 2.9 Statistical analysis

Continuous data were presented as mean ± SD. The differences between groups were compared using the two‐tailed unpaired Student *t* test. The *χ* ^2^ test or Fisher's exact test was employed to assess the correlation between the cell cycle gene‐based subtypes and clinical characteristics of HCC patients. The correlation between normally distributed variables was evaluated using Pearson's correlation test. *p* < 0.05 and |correlation coefficient (R)| > 0.3 indicate a significant correlation. All statistical tests were conducted with R (https://www.r‐project.org) and GraphPad Prism software (version 5.03; GraphPad Software Inc.). The Kaplan–Meier method was applied, and the survival of groups was compared using the log‐rank test. Findings with two‐sided *p* < 0.05 were deemed statistically significant.

Identification of differentially expressed genes (DEGs), DEGs enrichment analysis, Gene Set Variation Analysis (GSVA), Tumor Immune Dysfunction and Exclusion (TIDE) algorithm, and TIMER2.0 database analysis are detailed in Appendix S1.

## 3 RESULTS

### 3.1 Identification of two clusters with different clinical outcomes using the NMF algorithm

**FIGURE 1:**
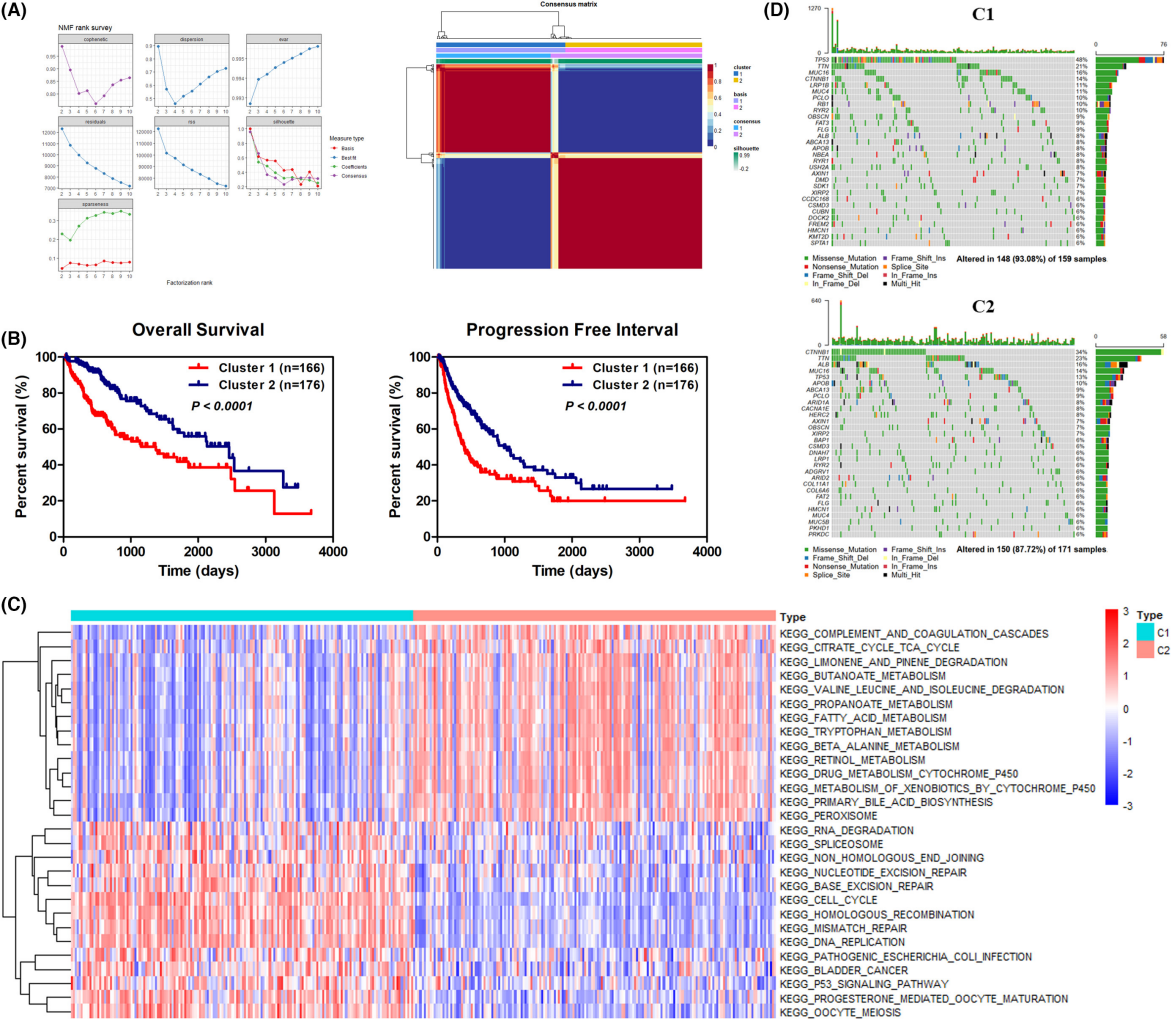
Identification of two clusters with different clinical outcomes and genomic variations in HCC via NMF algorithm and somatic mutation analysis. (A) Consensus map of non‐negative matrix factorization (NMF) clustering. (B) Kaplan–Meier survival curves of patients with C1 and C2 subtype cancers, for overall survival (OS) and progression‐free interval (PFI); *p‐*values were determined using the log‐rank test. (C) Heatmap of top 28 differentially enriched molecular pathways between the two subtypes. Red represents high activity while green represents low activity. (D) Somatic mutation analyses showing the correlations between tumor mutations and molecular subtypes of C1 and C2. Cluster 1, C1; Cluster 2, C2.

The NMF algorithm was applied to identify the molecular subtypes of HCC patients based on the 578 cell cycle‐related genes. HCC patients in the TCGA‐LIHC cohort were categorized into Cluster 1 and Cluster 2 molecular subtypes, according to the dispersion, cophenetic, and silhouette indicators (Figure 1A). As displayed in Figure 1B, Kaplan–Meier survival analysis indicated that Cluster 1 was markedly related to worse OS and shorter progression‐free interval (PFI). The median OS time of HCC patients in Cluster 1 was remarkably shorter compared with Cluster 2 (46 vs. 83 months, *p* < 0.001), and the median PFI time of Cluster 1 was markedly shorter than that of Cluster 2 (13 vs. 33 months, *p* < 0.001), as shown in Table S2. Next, the clinical characteristics of the two clusters were compared. Significant differences were observed for tumor histological grade and pTNM stages, as shown in Table S3. The prognostic significance of the two clusters in the context of various clinicopathological variables was evaluated by multivariable‐adjusted Cox regression analyses. The results revealed that HCC patients in Cluster 2 had significant better OS (HR = 0.51, *p* = 0.002) and PFI (HR = 0.62, *p* = 0.004) when compared to Cluster 1 (Table 1).

**TABLE 1:** Associations of molecular subtypes and clinicopathologic variables with overall survival (OS) and progression‐free survival (PFS), assessed using multivariable‐adjusted Cox regression.

Variables	Overall survival			Disease/Progression‐free survival		
	HR (95% CI)	*p*	*p*_trend_	HR (95% CI)	*p*	*p*_trend_
Molecular subtype						
C1	1 (reference)			1 (reference)		
C2	0.51 (0.33–0.77)	**0.002**		0.62 (0.44–0.86)	**0.004**	
Age (as continuous)	1.01 (0.99–1.03)	0.293		1.00 (0.99–1.01)	0.557	
Sex						
Male	1 (reference)			1 (reference)		
Female	1.11 (0.74–1.67)	0.616		0.93 (0.66–1.30)	0.652	
Histologic grade						
G1	0.92 (0.49–1.72)	0.791	0.599	1.03 (0.63–1.69)	0.906	0.994
G2	1 (reference)			1 (reference)		
G3	0.93 (0.60–1.45)	0.754		1.04 (0.74–1.48)	0.815	
G4	1.79 (0.69–4.67)	0.233		0.96 (0.38–2.43)	0.927	
pTNM stage						
I	1 (reference)		**<0.001**	1 (reference)		**<0.001**
II	1.49 (0.88–2.54)	0.141		1.89 (1.27–2.79)	**0.002**	
III	2.86 (1.81–4.52)	**<0.001**		2.47 (1.69–3.61)	**<0.001**	

*Note*: Associations of survival with molecular subtype and clinicopathologic factors were assessed using multivariable Cox proportional hazards regression with mutual adjustment for these factors. Bold values indicate statistical significance.

Abbreviations: C1, cluster 1; C2, cluster 2; CI, confidence interval; HR, hazard ratio.

### 3.2 Cluster 1 exhibits hyperactivated cell cycle program and frequent *TP53* mutation

To explore the underlying mechanisms related to the cell cycle gene‐based classification of HCC, we first identified 1202 differentially expressed genes (DEGs), including 386 downregulated and 816 upregulated genes, in Cluster 1 (Figure S2). Then, GSVA analysis was performed and differentially enriched molecular pathways were identified, including 14 and 56 pathways positively correlated with Cluster 1 and Cluster 2, respectively (Figure 1C; Table S4). Briefly, Cluster 1 was shown to be correlated with p53, DNA replication, and cell cycle pathways, whereas Cluster 2 was mainly correlated with molecular pathways related to metabolism, such as primary bile acid biosynthesis, fatty acid metabolism, and citrate cycle. Somatic mutation analysis revealed that each subtype possessed specific mutated genes. As shown in Figure 1D, *TP53* (48%) and *CTNNB1* (34%) were the most frequently mutated genes in Cluster 1 and Cluster 2, respectively. Taken together, these findings uncovered that activated cell cycle program and frequently *TP53* mutation were observed in Cluster 1 and might be associated with unfavorable clinical outcomes of HCC.

### 3.3 Construction and validation of immune gene signature for prognostic prediction of HCC

**FIGURE 2:**
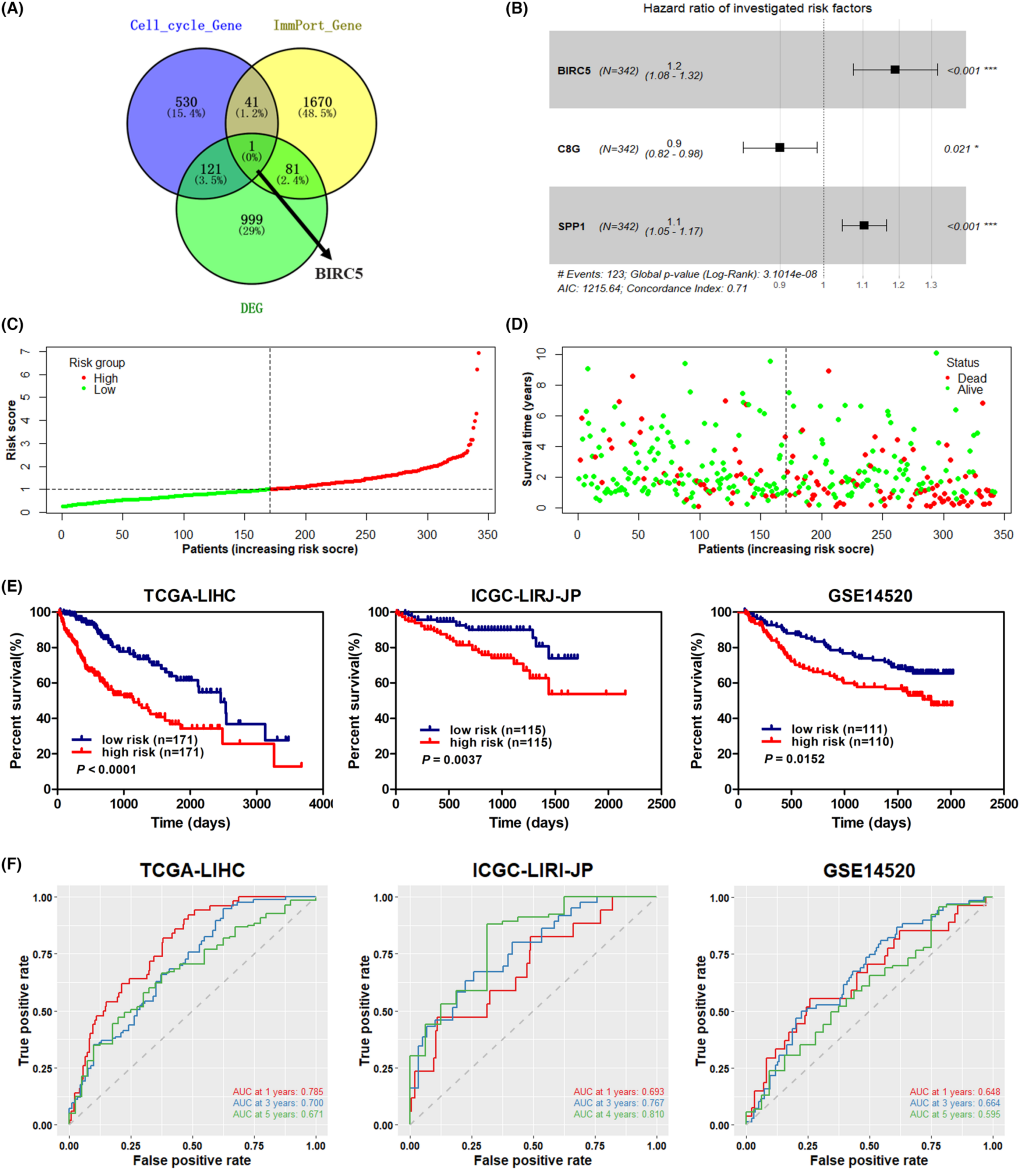
Construction of a prognostic risk model based on the two clusters. (A) Veen diagram showing the number of overlapped genes among the differentially expressed genes (DEGs), immune genes from the ImmPort database, and the cell cycle gene set. (B) Hazard ratios of investigated risk factors including *BIRC5*, *SPP1*, and *C8G*. (C) RS of each sample; red spot and green represents high‐risk group and low‐risk group, respectively. (D) survival status according to the RS of each sample; red spot and green represents dead and live, respectively. (E) Kaplan–Meier survival curves based on the 3‐gene‐signature risk model in the TCGA‐LIHC, LIRI‐JP, and GSE14520 datasets; *p*‐values were determined using the log‐rank test. (F) time‐dependent receiver operating characteristic (ROC) curves for the TCGA‐LIHC, LIRI‐JP, and GSE14520 datasets. RS, risk score.

Among the 1202 DEGs, 82 genes were immune‐related genes extracted from the Immport database (Figure 2A). A total of 24 immune genes were selected for the construction of RS model in all enrolled TCGA‐LIHC samples. A prognostic RS model composing of *BIRC5*, *SPP1*, and *C8G* was constructed via the LASSO algorithm. The results showed that the higher expression of *BIRC5* or *SPP1* implied a worsen prognosis of HCC (Figure 2B). Moreover, the number of deaths in the TCGA‐LIHC cohort was increased with increasing RS values (Figure 2C,D). To confirm and validate the performance of the prognostic RS model, Kaplan–Meier analysis was conducted to evaluate the association between OS and the 3‐gene‐signature risk model in TCGA‐LIHC and the validation datasets including GSE14520 and ICGC (LIRI‐JP). It was found that patients with a higher RS had shorter OS time (Figure 2E). Furthermore, the reliability of the prognostic RS model was evaluated by time‐dependent ROC curves. As shown in Figure 2F, the AUCs were 0.785, 0.700 and 0.671 for 1‐, 3‐, and 5‐year survival, respectively, in the TCGA‐LIHC training set. In the ICGC (LIRI‐JP) validation cohort, the AUCs were 0.693, 0.767, and 0.810 for 1‐, 3‐, and 4‐year survival, respectively. In the GSE14520 cohort, the AUCs were 0.648, 0.664, and 0.595 for 1‐, 3‐, and 5‐year survival, respectively. These results demonstrated a great potential of the constructed RS in estimating the overall survival of HCC patients.

### 3.4 Cluster 1 possesses higher MDSCs and is not sensitive to ICI therapies

**FIGURE 3:**
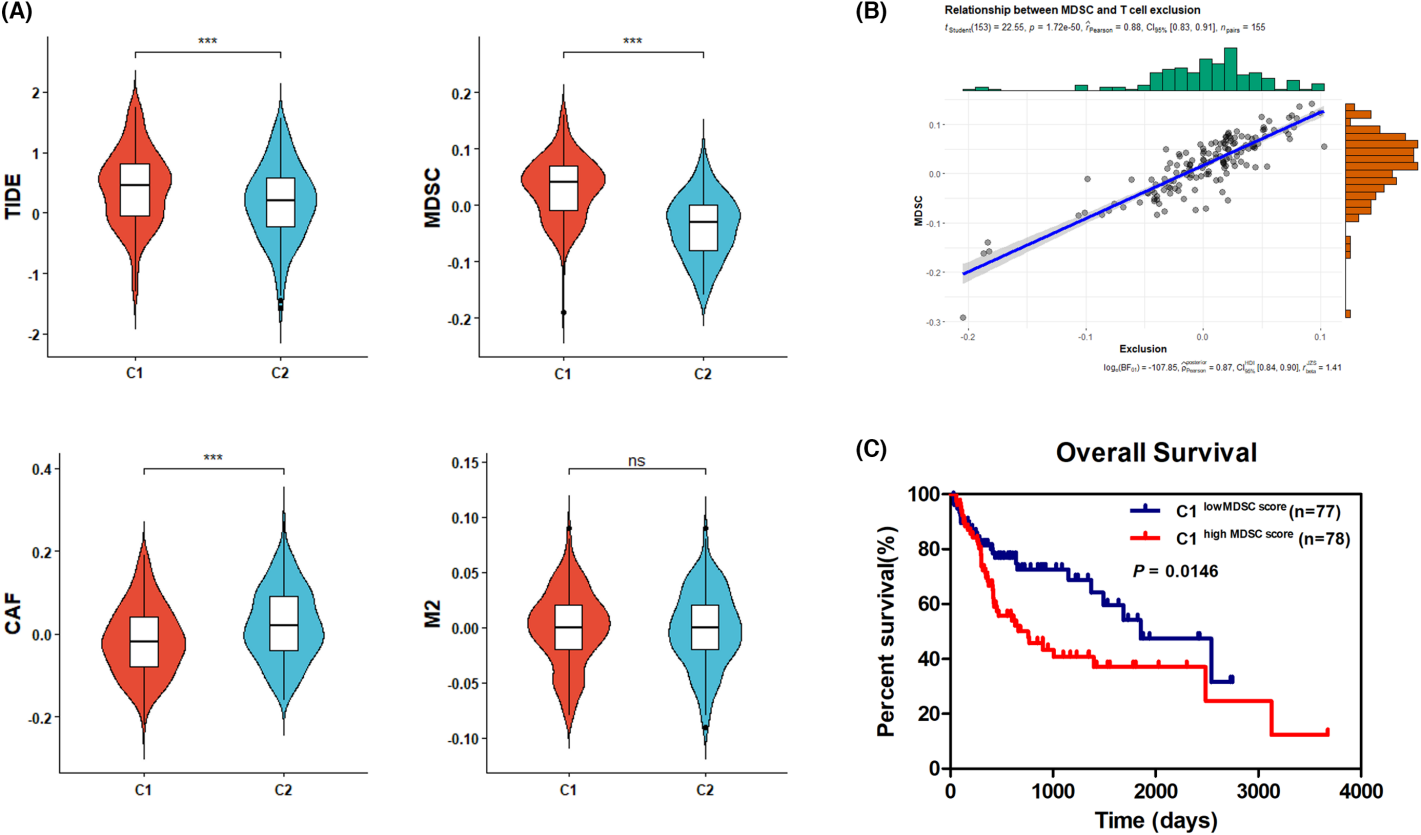
Distinct immune profile patterns between the two clusters. (A) Comparisons of the TIDE prediction and MDSCs, CAF, M2 scores between the C1 and C2. Data were analyzed using *t* test. **p < 0.01; ***p < 0.001. (B) Correlation of MDSCs scores and T cell exclusion scores in C1. (C) Kaplan–Meier survival curves of patients in C1 with high and low MDSCs score; the *p‐*value was determined using the log‐rank test. C1, Cluster 1; C2, Cluster 2.

Genomic alterations have been proposed to play potential roles in modulating tumor immunity and immune infiltration patterns.^32^ Then, the differences in immune profiles between the two clusters were further investigated. The M2 subtype of tumor‐associated macrophages, cancer‐associated fibroblasts (CAFs) and MDSCs are three cell types that suppress T cell infiltration in tumors.^33^ By utilizing the Tumor Immune Dysfunction and Exclusion (TIDE) algorithm,^34^ MDSC, CAF and M2 intratumor infiltration scores were calculated in patients from TCGA‐LIHC cohort. Multivariable‐adjusted Cox regression analyses revealed that among the three immune suppressive cells, the MDSC infiltration score was markedly related to OS and PFI like pTNM stages (Table 2), suggesting intratumor MDSC infiltration level was a significant prognostic factor for predicting the clinical outcomes of HCC patients. Notably, our results also revealed that patients in Cluster 1 had a significantly higher TIDE prediction score accompanied by a higher MDSCs infiltration score when compared to those in Cluster 2 (Figure 3A). Additionally, MDSC infiltration score was positively correlated with T‐cell exclusion score in C1 (Figure 3B) and survival analysis revealed that patients in Cluster 1 with a higher MDSCs score had shorter OS time compared to those with a lower MDSCs score (*p* = 0.0146) (Figure 3C). Collectively, these results revealed that intratumor infiltration of MDSCs level was an independent prognostic factor for HCC and patients in Cluster 1 possessed higher TIDE and MDSCs scores, which might account for the insensitivity of C1 to ICI therapies.

**TABLE 2:** Associations of TIDE scores and clinicopathologic variables with overall survival (OS) and progression‐free survival (PFS), assessed using multivariable‐adjusted Cox regression.

Variables	Overall survival			Progression‐free interval		
	HR (95% CI)	*p*	*p*_trend_	HR (95% CI)	*p*	*p*_trend_
TIDE score of MDSC						
Low	1 (reference)			1 (reference)		
High	1.68 (1.08–2.61)	**0.021**		1.54 (1.07–2.20)	**0.019**	
TIDE score of CAF						
Low	1 (reference)			1 (reference)		
High	0.78 (0.52–1.18)	0.243		0.75 (0.54–1.05)	0.096	
TIDE score of M2						
Low	1 (reference)			1 (reference)		
High	0.87 (0.58–1.33)	0.524		0.82 (0.59–1.15)	0.256	
Age (as continuous)	1.01 (0.99–1.02)	0.427		1.00 (0.98–1.01)	0.465	
Sex						
Male	1 (reference)			1 (reference)		
Female	1.15 (0.77–1.73)	0.488		0.91 (0.65–1.28)	0.586	
Histologic grade						
G1	0.83 (0.44–1.55)	0.551	0.558	0.94 (0.57–1.54)	0.801	0.987
G2	1 (reference)			1 (reference)		
G3	0.95 (0.61–1.47)	0.803		1.03 (0.73–1.46)	0.875	
G4	1.78 (0.67–4.70)	0.245		0.95 (0.37–2.41)	0.907	
pTNM stage						
I	1 (reference)		**<0.001**	1 (reference)		**<0.001**
II	1.51 (0.88–2.56)	0.132		1.90 (1.28–2.81)	**0.001**	
III	3.13 (1.98–4.96)	**<0.001**		2.59 (1.78–3.79)	**<0.001**	

*Note*: Associations of survival with molecular subtype and clinicopathologic factors were assessed using multivariable Cox proportional hazards regression with mutual adjustment for these factors. Bold values indicate statistical significance.

Abbreviations: CAF, cancer‐associated fibroblast; CI, confidence interval; HR, hazard ratio; M2, macrophage type; MDSC, myeloid‐derived suppressor cell; TIDE, tumor immune dysfunction and exclusion.

### 3.5 Concordant overexpression of Birc5 and higher MDSC infiltration is correlated with poor prognosis of HCC

**FIGURE 4:**
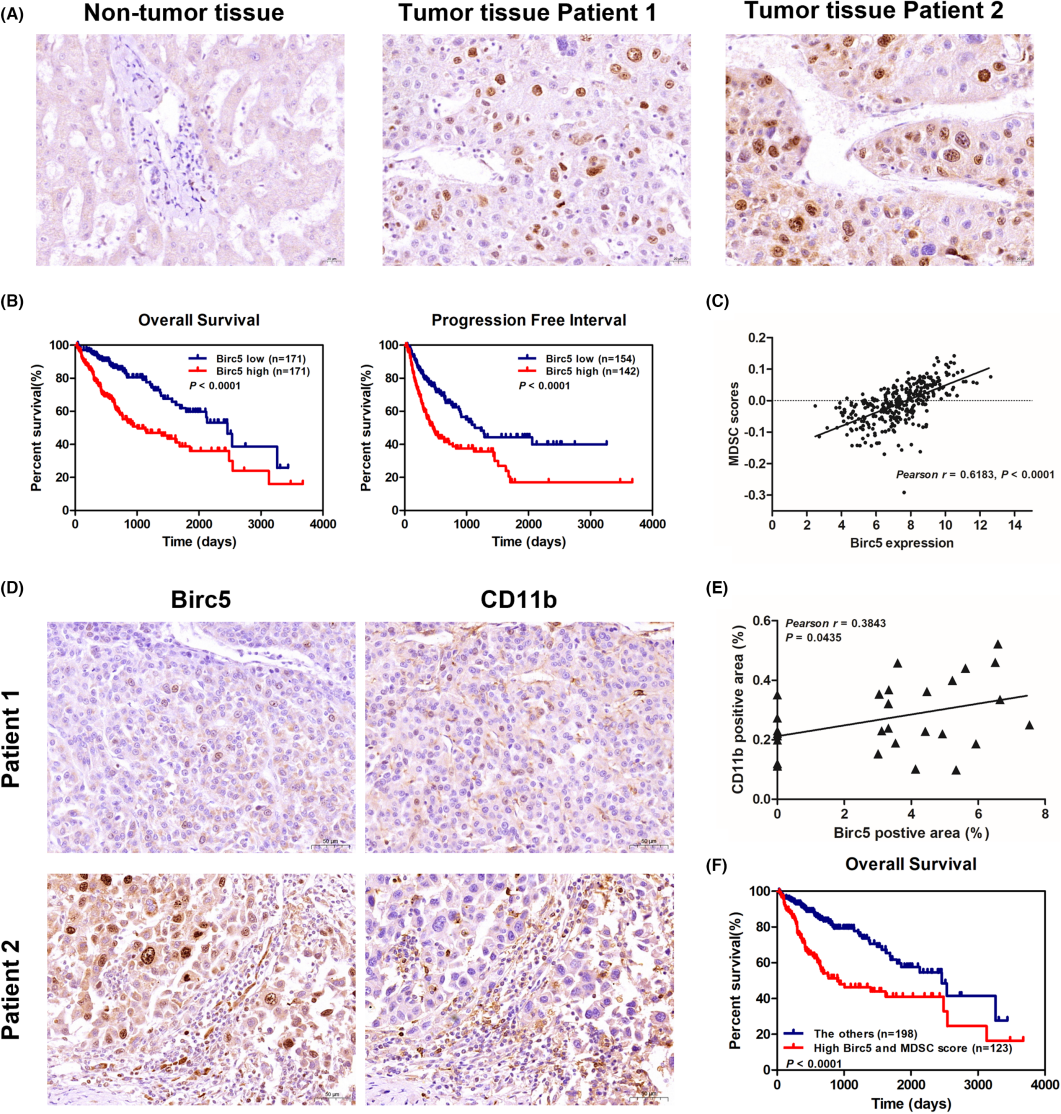
Birc5 expression was positively associated with the infiltration of MDSCs in HCC. (A) Representative immunostaining images of Birc5 expression in tumor and non‐tumor tissues of HCC patients (×40 magnification, black bar down right 20 μm). (B) Kaplan–Meier survival curves of HCC patients in TCGA‐LIHC cohort with high and low Birc5 expression regarding overall survival and progression‐free interval; the *p*‐values were calculated using the log‐rank test. (C) Correlation of Birc5 expression with MDSCs score in patients from TCGA‐LIHC cohort. (D) Representative immunostaining images for Birc5 and CD11b in HCC tumor tissues (×20 magnification, black bar down right 50 μm). (E) Correlation of Birc5 with CD11b staining in HCC tumor tissues. (F) Kaplan–Meier survival curves associated with high *BIRC5* expression and MDSCs infiltration score versus the others in HCC from TCGA‐LIHC cohort.

**FIGURE 5:**
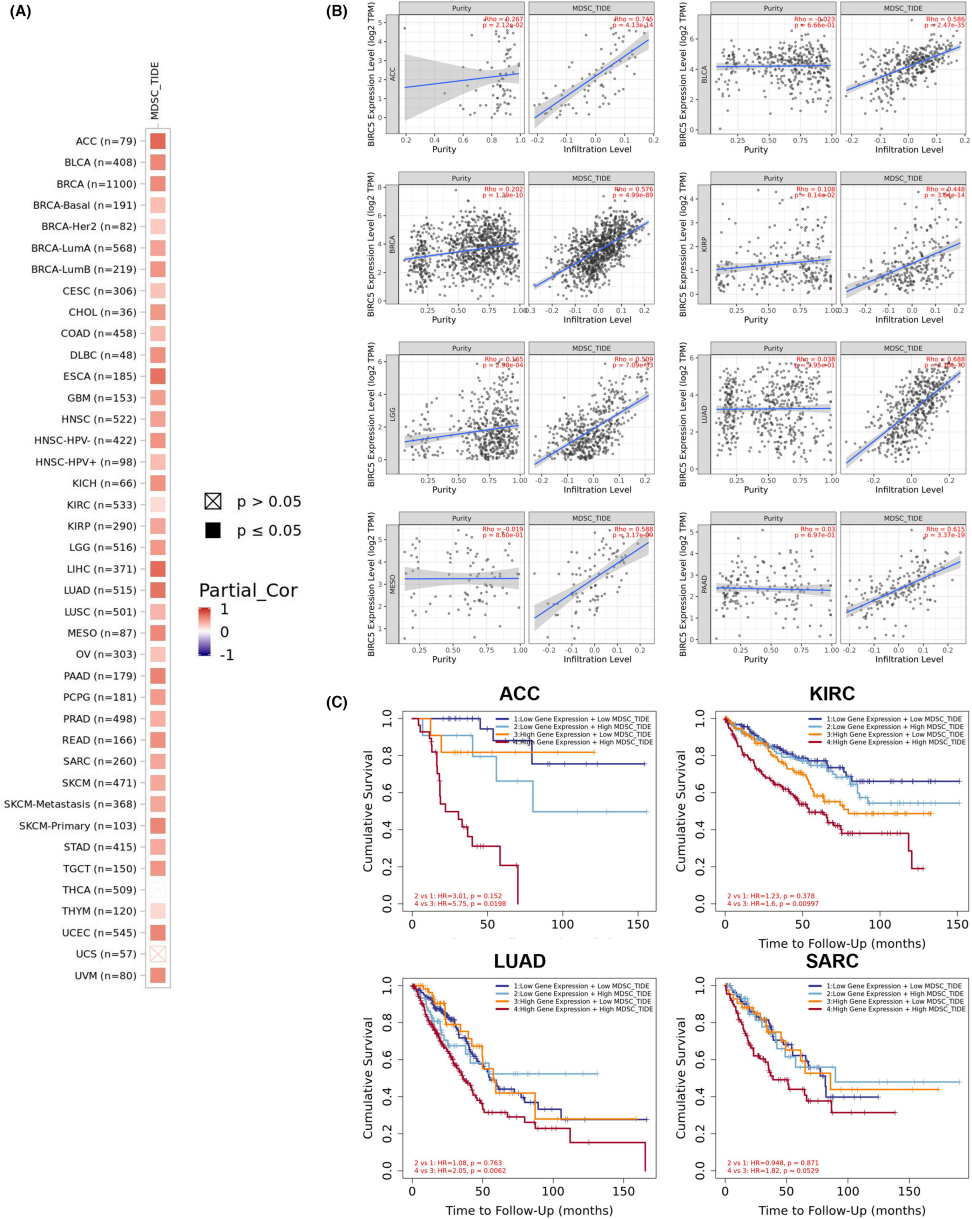
Concordant high expression of Birc5 and intratumor infiltration level of MDSCs correlates with poorer prognosis of patients in multiple cancers. (A) Heatmap showing the correlation between Birc5 expression and myeloid‐derived suppressor cells (MDSCs) scores in multiple cancers. (B) Correlations of *BIRC5* expression with tumor purity and MDSCs filtration levels in patients with indicated cancer based on the TIMER and CIBERSORT databases. (C) Kaplan–Meier survival curves of patients with high and low *BIRC5* expressions and MDSC infiltration scores in adrenocortical carcinoma (ACC), kidney renal clear cell carcinoma (KIRC), lung adenocarcinoma (LUAD), and sarcoma (SARC).

Among the 3 prognostic genes, *BIRC5* is the only gene belonging to cell cycle‐related and immune‐related genes (Figure 2A). Immunohistochemical staining confirmed that Birc5 was overexpressed in the nucleus of HCC tumor cells (Figure 4A). Survival analysis revealed that patients with higher Birc5 expression had shorter OS time and DFI in the TCGA‐LIHC cohort (Figure 4B). We further created a nomogram combining Birc5 and clinicopathological information using the TCGA‐LIHC cohort (C index, 0.71), and the calibration curves illustrated that the predicted and actual survival had a good consistency (Figure S3). In addition, Birc5 expression was positively correlated with MDSC infiltration score in TCGA‐LIHC cohort (Figure 4C). In HCC tumor tissue samples, CD11b was used to indicate MDSC infiltration level, and a positive correlation between CD11b and Birc5 staining was observed (Figure 4D,E). Survival analysis indicated that the patients with Birc5 overexpression and MDSC infiltration score had shorter OS time when compared to those with HCC. By utilizing TIMER dataset, it was observed that *BIRC5* expression was positively correlated with MDSC infiltration score in multiple cancers (Figure 5A,B), and survival analysis demonstrated that patients with higher *BIRC5* expression and MDSC infiltration score had shorter OS time in sarcoma, lung adenocarcinoma, kidney renal clear cell carcinoma, and adrenocortical carcinoma (Figure 5C). Therefore, these findings implied that *BIRC5* might be used as a biomarker associated with MDSC infiltration in multiple cancers, including HCC, and concordant overexpression of Birc5 and higher MDSC infiltration was correlated with poor prognosis of several tumors.

### 3.6 Birc5 regulates the immune microenvironment of HCC

**FIGURE 6:**
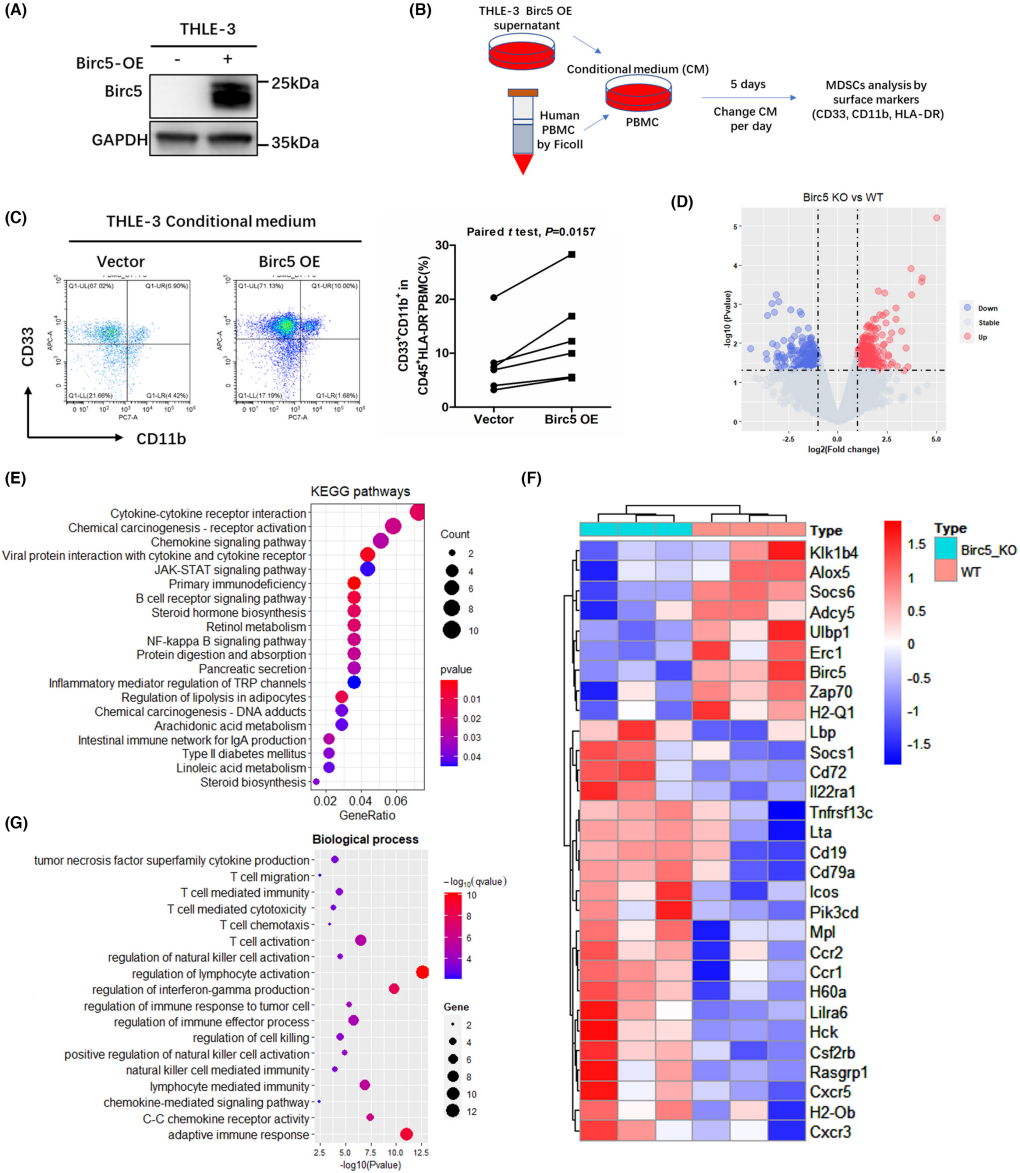
Potential mechanism by which Birc5 promoted MDSC amplification. (A) Western blot analyses of Birc5 expression in THLE‐3 cells treated with control or Birc5‐overexpressing lentivirus. (B) Schematic diagram. Human peripheral blood mononuclear cells (PBMCs) from healthy donors were cultured with the conditional medium of THLE‐3 cells with or without Birc5 overexpression, followed by MDSC phenotypic analysis. (C) The representative flow cytometry dot plots, the quantification and comparison of the percentages of CD45^+^HLA^−^DR^−^CD33^+^CD11b^+^MDSCs in PBMCs from 6 human donors treated with the conditional medium of THLE‐3 cells with or without Birc5 overexpression in vitro. (D) Volcano plot of DEGs upon Birc5 depletion in a transgenic animal model of liver cancer. (E) Dot plot of KEGG pathway of the DEGs upon Birc5 depletion. (F) Heatmap of immune‐related DEGs upon Birc5 depletion. (G) Dot plot of Biological Process of the immune‐related DEGs upon Birc5 depletion.

Reportedly, Birc5 inhibits apoptosis and promotes proliferation, invasion, migration, and drug resistance in HCC cells.^35^, ^36^, ^37^ To further investigate whether Birc5 contributes to MDSC infiltration, the expression of Birc5 was evaluated in the normal human hepatocyte cells THLE‐3 and human liver cancer cells Huh7. It was found that Birc5 was highly expressed in Huh7 when compared to THLE‐3 (Figure S4). Subsequently, THLE‐3 cells with stable Birc5 overexpression were established using lentivirus, and the efficacy of overexpression was detected by western blotting (Figure 6A). Next, PBMCs from healthy human donors were cultured in the conditioned medium collected from the supernatant of THLE‐3 cells with stable Birc5 overexpression. The flowchart of cultivation strategy is shown in Figure 6B. Flow cytometry showed that the supernatant of Birc5‐overexpressing THLE‐3 cells significantly promoted the amplification of MDSCs (Figure 6C). By analyzing the whole‐genome expression profile of mouse liver cancer with or without *BIRC5* depletion (GSE64804), 343 DEGs were obtained (Figure 6D). KEGG pathway enrichment analysis revealed that DEGs related to *BIRC5* depletion were enriched in chemokine, cytokine–cytokine receptor interaction, T‐cell receptor, JAK–STAT, and NF‐kappa B pathways (Figure 6E). Heatmap showed that 30 immune‐related genes enriched in these pathways were dysregulated upon *BIRC5* depletion (Figure 6F). Gene ontology enrichment analysis indicated that these 30 genes were remarkably enriched in adaptive immune response, chemokine‐mediated signaling pathway, lymphocyte‐mediated immunity, natural killer cell‐mediated immunity, interferon‐gamma production, T‐cell activation, and T‐cell‐mediated cytotoxicity (Figure 6G). Taken together, these findings demonstrate that Birc5 plays a vital role in modulating immune profile and response in HCC. The underlying mechanism might be associated with the function of Birc5 in recruiting MDSCs and regulating the expression of cytokine or chemokine, as well as inhibiting the activation of antitumor immune cells.

## 4 DISCUSSION

Growing evidence from preclinical studies and clinical trials indicates that the hyperactivation of cell cycle program in tumor cells might confer protection from immune surveillance.^38^ Therefore, identification and validation of new biomarkers related to cell cycle program may offer novel mechanistic insights and therapeutic targets to improve the immune responses of HCC to immunotherapy.

In this work, based on the expression profiles of genes related to cell cycle program, we stratified HCC from the TCGA‐LIHC cohort into two clusters (Cluster 1 and Cluster 2) using the NMF method. Multivariable‐adjusted Cox regression analysis showed that the cell cycle gene‐based classification like pTNM stage was a significant prognostic factor for predicting the clinical outcome of HCC patients. Briefly, HCC patients in Cluster 1 presented worse OS and PFI compared with those in Cluster 2. GSVA analysis revealed that genes related to p53, DNA replication, and cell cycle pathways were significantly enriched in the Cluster 1. Genomic variation analysis showed that *TP53* mutation was more frequently observed in Cluster 1, whereas Cluster 2 was characterized by *CTNNB1* mutation. Previously, *TP53* mutation has been reported to be a potential prognostic marker of several cancers, including HCC, lung cancer, clear cell renal cell carcinoma, and acute myeloid leukemia.^39^ Mechanistically, *TP53* mutation has been reported to play vital roles in determining tumor immune profile and PD‐L1 expression in lung adenocarcinoma.^17^ Mutant *TP53* suppressed innate immune signaling and promoted immune evasion.^40^ Therefore, these results imply that activated cell cycle program may account for unfavorable clinical outcomes and affect the tumor immune profile of HCC patients.

Next, we further investigated the differences in immune cell infiltration patterns between Cluster 1 and Cluster 2. TIDE algorithm was used to predict the therapeutic responses to ICIs, and the results demonstrated that HCC patients in Cluster 1 exhibited a higher TIDE score compared with those in Cluster 2, implying a worse response of patients in Cluster 1 to ICI therapy. Among three immunosuppressive cells (MDSCs, CAF, and M2), MDSCs was predominant in Cluster 1 and multivariable‐adjusted Cox regression analysis revealed that the MDSC infiltration score was a significant prognostic factor associated with OS and PFI. It has been well known that MDSCs are a pivotal cell population that contributes to the formation of tumor‐induced immunosuppression through restricting T‐cell accumulation in the vicinity of tumor cells and promoting tumor cell evasion from the immune system.^33^, ^41^ Moreover, the positive correlation between MDSC infiltration and T‐cell exclusion has been proven to be a potential mechanism corresponded to the failure of antitumor therapy including ICI in several tumors.^20^, ^33^, ^34^ Zhou et al^42^ showed the CD11b^+^CD33^+^HLA‐DR^−^MDSCs was upregulated in both peripheral blood and intratumor tissues and could inhibit CD8^+^ T‐cell proliferation in patients with HCC. Consistent with their findings, we found that MDSCs were positively correlated with T‐cell exclusion score and survival analysis revealed that HCC patients with a higher MDSC score had shorter OS time compared to those with a lower MDSC score in Cluster 1. Taken together, these findings imply that a higher level of MDSC infiltration may account for poor prognosis and immune therapy failure in HCC patients in Cluster 1, probably through inhibiting the intratumor accumulation of cytotoxic T cells.

To characterize the cell cycle‐based classification of HCC, we constructed a three‐gene prognostic model, including *BIRC5*, *C8G*, and *SPP1*, which had strong robustness and a stable predictive performance in the datasets from two different cohorts. Notably, among the three risk genes, *BIRC5* was the only gene positively correlated with the infiltration of MDSCs in HCC. Birc5, also known as Survivin, is significantly upregulated in several tumors.^43^, ^44^ It has been shown that Birc5 is a poor prognostic marker of HCC by promoting cancer cell proliferation, inducing angiogenesis, and attenuating the sensitivity of tumor cells to radiotherapy and chemotherapy.^45^ Recently, the relationships among Birc5, immune cell infiltration, and the clinical outcome of multiple cancers have been reported.^43^ It was found that Birc5 expression was related to immune cell infiltration in the TME of several tumors. However, the underlying mechanism is less reported in HCC. Our results showed that Birc5 expression was positively correlated with MDSC infiltration level and CD11b expression (a MDSC marker) in HCC as well as in multiple cancers. Of note, patients with upregulated expression of Birc5 and higher level of MDSC infiltration exhibited worse clinical outcomes in HCC and other cancers such as KIRC, LUAD, and SARC. Taken together, we speculate that tumor‐intrinsic expression of Birc5 may contribute to the infiltration of MDSCs in tumor immune microenvironment.

To further investigate the underlying mechanism, Birc5 was overexpressed by lentivirus in human hepatocytes in vitro. By treating human PBMC with the supernatant of hepatocytes with Birc5 overexpression, our findings demonstrated that hepatocellular Birc5 overexpression promoted immunosuppressive CD11b^+^CD33^+^HLA‐DR^−^MDSC expansion. RNA‐seq revealed that Birc5 overexpression could affect the expression levels of several inflammatory factors or chemokines, such as IL1B, TGFBR2, MME, CCL2, and CXCR4, in hepatocellular cells, and the genes related to Birc5 overexpression were enriched in pathways related to hematopoietic cell lineage, TGF‐β, cytokine–cytokine receptor interaction, p53, and Wnt signaling (data not shown). Among these genes, *IL1B*, which encodes IL‐1β, has been shown to promote the infiltration of polymorphonuclear MDSCs in renal cell carcinoma.^46^ Furthermore, we analyzed the microarray data of an animal liver cancer model with or without *BIRC5* depletion.^24^ The findings revealed that the DEGs upon *BIRC5* depletion were enriched in chemokine‐mediated signaling pathway, lymphocyte‐mediated immunity, natural killer cell‐mediated immunity, interferon‐gamma production, T‐cell activation, and T‐cell‐mediated cytotoxicity. Moreover, Li et al^24^ showed that Birc5 deficiency could lead to increased CD3^+^ T lymphocytes in tumor sites and remarkable HCC suppression. Altogether, these findings suggest that Birc5 may be a regulator of antitumor immune responses by inhibiting the activation of natural killer cells and T cells.

Birc5 expression was related to immune cell infiltration in the tumor microenvironment of several tumors. However, the underlying mechanism is less reported. In this study, through bioinformatics analyses and in vitro experiments, we showed that activated cell cycle program was associated with immunosuppression, weakened immunotherapy efficiency, and worse prognosis in HCC patients, and that Birc5 was a potential biomarker and inducer of intratumor infiltration of MDSCs. MDSCs are a heterogeneous group of pathologically activated immature cells and can be further identified into two different subsets: polymorphonuclear‐ (PMN‐) and monocytic‐ (M‐) MDSCs.^47^ In HCC, the roles of M‐MDSC and PMN‐MDSCs in suppressing antitumor immunity remain controversial. For instance, increased accumulation of M‐MDSCs in mice liver tissues with liver fibrosis was significantly correlated with decreased infiltration of lymphocytes and increased tumorigenicity, instead of PMN‐MDSCs.^48^ Nevertheless, Zhou et al^42^ revealed that PMN‐MDSCs were able to inhibit tumor infiltration of IFN‐γ+TNF‐α+CD8^+^ T lymphocytes through the expression of Arg‐1. Therefore, to clearly illustrate the immune‐relevant function of Birc5 in regulating HCC tumor immune microenvironment, we still need to further answer which type of MDSCs contribute to the immunosuppressive tumor microenvironment and by which signaling pathways, cytokines, and chemokines, Birc5 impacts the amplification and infiltration of MDSCs in tumor tissue, as well as the clinical significance of targeting Birc5 in immunotherapy of HCC. Cancer therapeutics targeting Birc5/survivin include survivin‐partner protein interaction inhibitors, survivin homodimerization inhibitors, *survivin* gene transcription inhibitors, *survivin* mRNA inhibitors, and survivin immunotherapy.^44^ YM155 is a small molecule strongly inhibiting Birc5 expression at both the protein and mRNA levels, and we plan to use YM155 for Birc5 inhibitory experiments in our future research.

There are some other HCC‐related markers of note as revealed by some important studies. Wang et al^9^ identified a novel eight‐gene (*PAM*, *NUP155*, *GOT2*, *KDELR3*, *PKM*, *NSDHL*, *ENO1*, and *SRD5A3*) signature associated with cell glycolysis to predict survival of HCC patients. Feng et al^11^ identified ARID1A as a prognostic biomarker associated with immune infiltrates in HCC: high ARID1A expression was associated with worse prognosis and low immune cell infiltration; decreasing ARID1A expression inhibited cell cycle, proliferation, migration, and invasion in HCC.

The main limitation of this study is the lack of further in vivo experiments to validate the effect of biomarker‐directed therapy. Further explorations should be focused on the underlying mechanisms by which Bric5 regulates the infiltration of different types of MDSCs (PMN‐MDSCs or M‐MDSCs) in the TME and on how they contribute to T‐cell exclusion or dysfunction in HCC.

## 5 CONCLUSIONS

Birc5 was a potential biomarker and inducer of intratumor infiltration of MDSCs, which led to T‐cell exclusion or dysfunction in tumor immune microenvironment, consequently resulting in reduced response to ICIs in HCC.

## AUTHOR CONTRIBUTIONS

**Yun Liu:** Conceptualization (equal); data curation (equal); formal analysis (equal); investigation (equal); methodology (equal); project administration (equal); resources (equal); validation (equal); visualization (equal); writing – original draft (equal). **Xi Chen:** Data curation (equal); formal analysis (equal); investigation (equal); methodology (equal); writing – review and editing (equal). **Wenwu Luo:** Data curation (equal); formal analysis (equal); investigation (equal); methodology (equal); writing – review and editing (equal). **Yufei Zhao:** Data curation (equal); formal analysis (equal); investigation (equal); methodology (equal); writing – review and editing (equal). **Björn Nashan:** Data curation (equal); formal analysis (equal); investigation (equal); methodology (equal); supervision (equal); writing – review and editing (equal). **Lei Huang:** Conceptualization (equal); data curation (equal); formal analysis (equal); investigation (equal); methodology (equal); project administration (equal); resources (equal); software (equal); supervision (equal); validation (equal); writing – review and editing (equal). **Xiaodong Yuan:** Conceptualization (equal); data curation (equal); formal analysis (equal); funding acquisition (equal); investigation (equal); methodology (equal); project administration (equal); resources (equal); software (equal); validation (equal); visualization (equal); writing – original draft (equal).

## FUNDING INFORMATION

This study was supported by the Fundamental Research Funds for the Central Universities (grant number WK9110000131 to XY) and the Anhui Provincial Natural Science Foundation (grant number 2108085MH256 to XY).

## CONFLICT OF INTEREST STATEMENT

The authors declare that they have no competing interests.

## ETHICS APPROVAL AND CONSENT TO PARTICIPATE

The study protocol involving human subjects was approved by the Ethics Committees of the First Affiliated Hospital of USTC (2021‐RE‐113). All participation gave written informed consent in accordance with the Declaration of Helsinki.

## Supporting information


Appendix S1.

## Data Availability

Sequencing data used during the current study are available from the corresponding author on reasonable request.
