# Supplementary material for: Identification and validation of Birc5 as a novel activated cell cycle program biomarker associated with infiltration of immunosuppressive myeloid‐derived suppressor cells in hepatocellular carcinoma
Source: Cancer Med. 2023 Jun 16;12(15):16370–85. doi: 10.1002/cam4.6271 (PMC10469657; doi:10.1002/cam4.6271)
Supplement: Supplementary file 1 — Appendix S1. [file CAM4-12-16370-s001.docx]

**Supplementary materials**

**Supplementary Methods**

**Supplementary Figure 1. Flowchart of the study.**

**Supplementary Figure 2. Volcano plot of differentially expressed genes (DEGs) between the two Clusters.**

**Supplementary Figure 3. (A)** Nomogram for predicting the probability of 1- and 5-year overall survival of HCC patients in the TCGA-LIHC cohort. **(B)** Calibration curves of 3‐year and 5‐year overall survival (OS) for HCC patients in the TCGA-LIHC cohort.

**Supplementary Figure 4. Western blot images showing Birc5 expression in THLE-3 and Huh7.**

**Supplementary Table 1.** Clinicopathological features of enrolled HCC patients for immunohistochemistry validation

**Supplementary Table 2.** Clinicopathological parameters of patients with LIHC by the two Clusters

**Supplementary Table 3.** Associations of Cluster 2 versus Cluster 1 with clinicopathologic factors, assessed using multivariable-adjusted logistic regression

**Supplementary Table 4.** Differentially enriched molecular pathways between the two Clusters

**Supplementary Methods**

***Identification of differentially expressed genes (DEGs) and DEGs enrichment analysis***

DEGs between the subtypes were analyzed using the limma package in R [1]. A total of 1202 DEGs (adjusted *P* value <0.05 and |log_2_FC| >1) were identified between C1 and C2. As for the mouse liver cancer dataset (GSE64804), DEGs (*P* value <0.05 and |log_2_FC| >0.5) between mouse liver cancers with and without *BIRC5* depletion were identified. The “clusterProfiler” package of the R software were used to perform Gene Ontology (GO) and Kyoto Encyclopedia of Genes and Genomes (KEGG) enrichment analyses of DEGs as previously described. Categories with *P* and q values of <0.05 were regarded as significantly enriched.

***Gene set variation analysis (GSVA)***

GSVA was applied to evaluate the enriched molecular pathways in the subtypes using the GSVA package in R[2]. Differential analysis of the enrichment scores of KEGG pathways between the two subtypes was performed using the limma package in R. KEGG pathways with |log_2_FC| >0.1 and adjusted *P* value <0.05 were considered as the significantly enriched molecular pathways.

***Tumor immune dysfunction and exclusion (TIDE) algorithm***

The TIDE algorithm was applied to evaluate the potential capability of tumor cells in tumor immune escape and the potential response of cancer cells to ICI treatment (<http://tide.dfci.harvard.edu/>) [3]. To perform the TIDE algorithm, the average value of the RNA sequencing data of all enrolled TCGA-LIHC samples was used as the normalized control. T exclusion and T dysfunction scores of TCGA-LIHC were downloaded from the TIDE dataset, which was available under the GNU Public License Version 3 (<http://tide.dfci.harvard.edu/>).

***TIMER2.0 database analysis***

TIMER2.0 is a comprehensive source that can explore the correlations between gene expression and infiltration of immune cells [4]. In this study, the correlations and survival analysis associated with BIRC5 gene expression and MDSCs infiltration levels in multiple cancers were explored using the TIMER2.0 online database.

**References**

1. Liu, Y., Y. Zhao, J. Fang, J. Fang, and X. Yuan, *Bioinformatics analysis of microenvironment-related genes associated with radioresistance in glioblastoma.* Transl Cancer Res, 2020. **9**(12): p. 7495-7504.

2. Hanzelmann, S., R. Castelo, and J. Guinney, *GSVA: gene set variation analysis for microarray and RNA-seq data.* BMC Bioinformatics, 2013. **14**: p. 7.

3. Jiang, P., S. Gu, D. Pan, J. Fu, A. Sahu, X. Hu, Z. Li, N. Traugh, X. Bu, B. Li, J. Liu, G.J. Freeman, M.A. Brown, K.W. Wucherpfennig, and X.S. Liu, *Signatures of T cell dysfunction and exclusion predict cancer immunotherapy response.* Nat Med, 2018. **24**(10): p. 1550-1558.

4. Li, T., J. Fu, Z. Zeng, D. Cohen, J. Li, Q. Chen, B. Li, and X.S. Liu, *TIMER2.0 for analysis of tumor-infiltrating immune cells.* Nucleic Acids Res, 2020. **48**(W1): p. W509-W514.


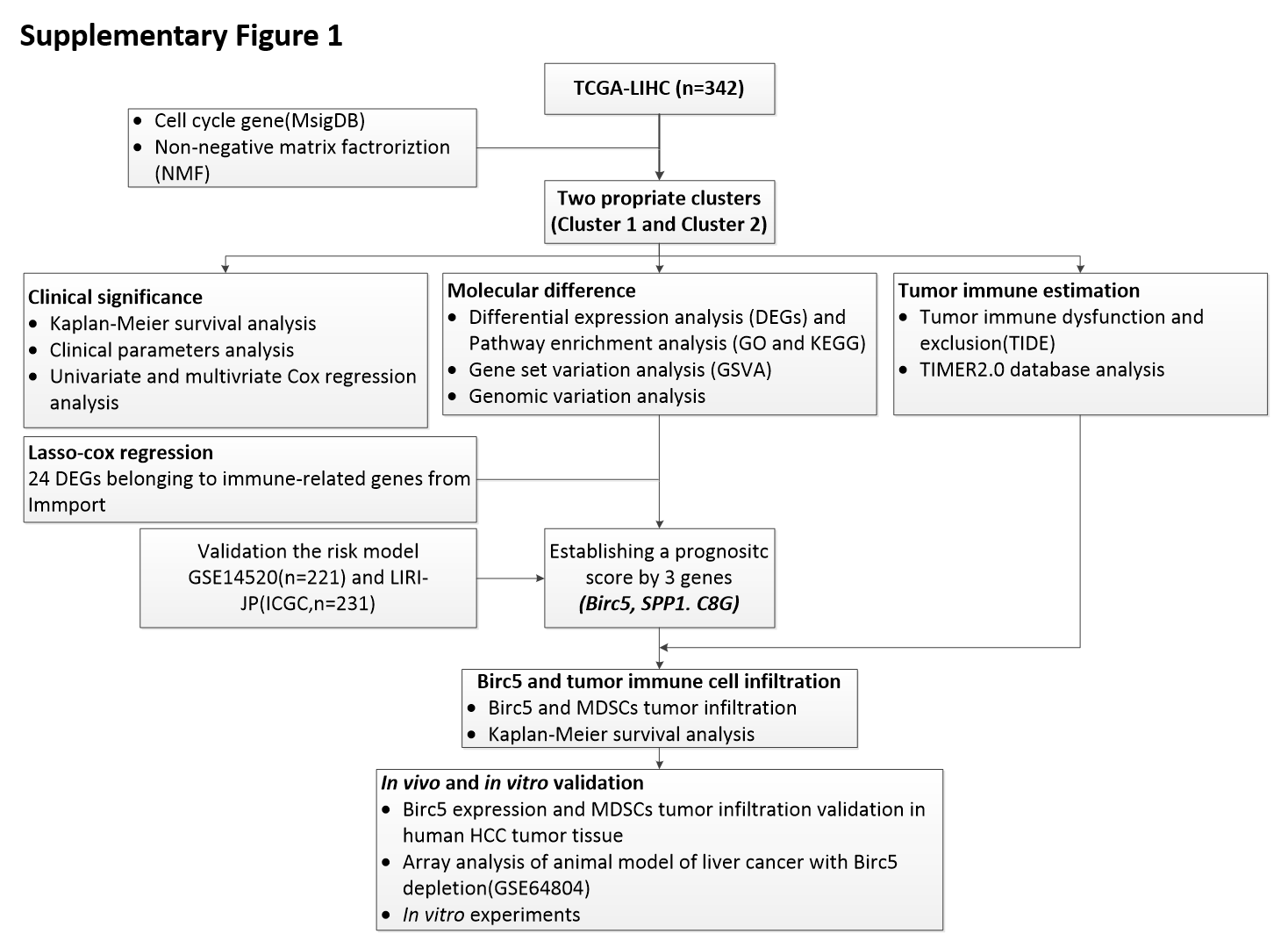


**Supplementary Figure 1. Flowchart of the study.**


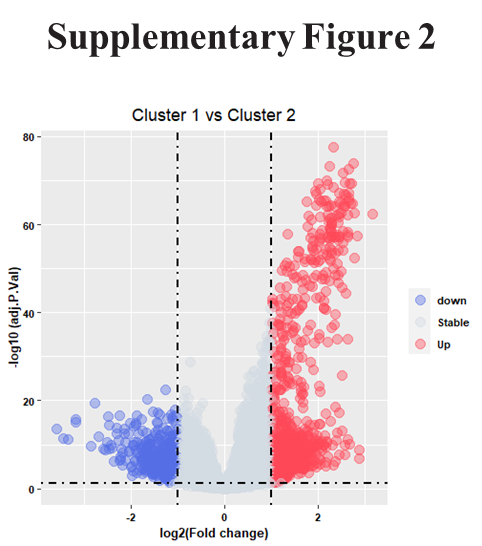


**Supplementary Figure 2.** Volcano plot of differentially expressed genes (DEGs) between the two Clusters.


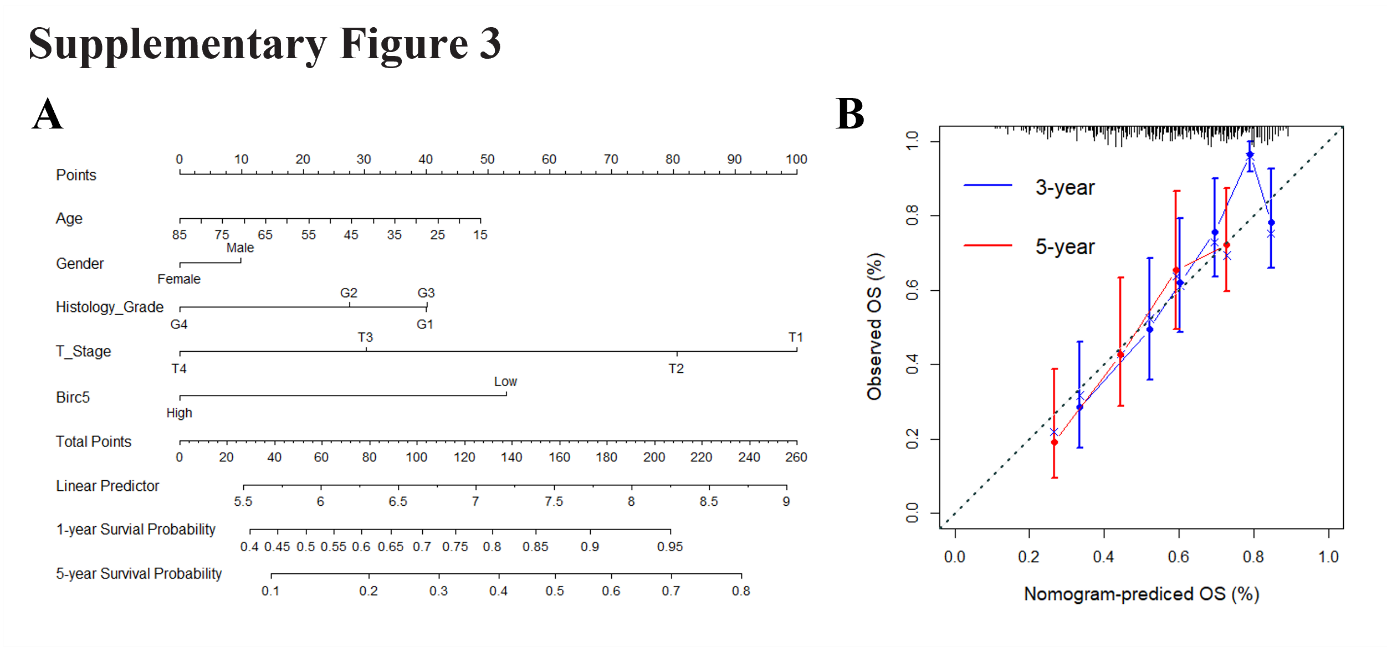


**Supplementary Figure 3.** **(A)** Nomogram for predicting the probability of 1- and 5-year overall survival of HCC patients in the TCGA-LIHC cohort. **(B)** Calibration curve of 3‐year and 5‐year overall survival (OS) for HCC patients in the TCGA-LIHC cohort.


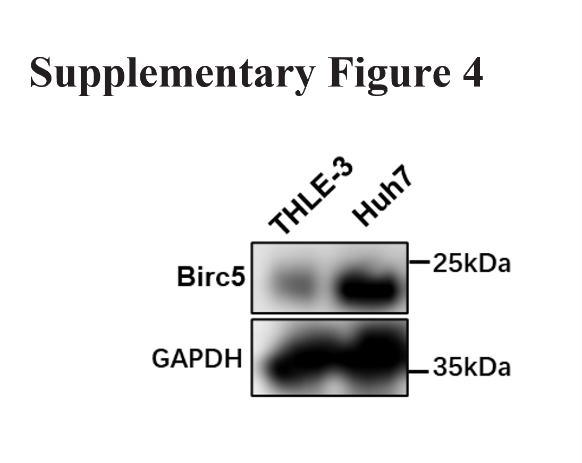


**Supplementary Figure 4. Western blot images showing Birc5 expression in THLE-3 and Huh7.**

**Supplementary Table 1.** Clinicopathological features of enrolled HCC patients for immunohistochemistry validation

| Clinicopathological features | Category | Value |
| --- | --- | --- |
| n |  | 28 |
| Age (years) | As continuous | 61±11 |
| Gender | Male | 21 (75) |
|  | Female | 7 (25) |
| Histological grade | G1 | 2 (7) |
|  | G2 | 21 (75) |
|  | G3 | 5 (18) |

Continuous variables are shown as mean ± standard deviation, and categorical variables are shown as count (percentage [%]).

**Supplementary Table 2.** Clinicopathological parameters of patients with LIHC by the two Clusters

| Variables | Overall (n=315) | Cluster 1 (n=154) | Cluster 2 (n=161) | *P* |
| --- | --- | --- | --- | --- |
| Age (years) | 59±13; 60 (51 to 68) | 58±12; 59 (50 to 66) | 59±14; 62 (52 to 69) | **0.046** |
| Sex, male | 218 (69) | 99 (64) | 119 (74) | 0.064 |
| Histologic grade |  |  |  | **<0.001** |
| G1 | 43 (14) | 12 (8) | 31 (19) |  |
| G2 | 152 (48) | 66 (43) | 86 (53) |  |
| G3 | 108 (34) | 67 (44) | 41 (25) |  |
| G4 | 12 (4) | 9 (6) | 3 (2) |  |
| pTNM stage |  |  |  | **0.009** |
| I | 159 (50) | 65 (42) | 94 (58) |  |
| II | 76 (24) | 40 (26) | 36 (22) |  |
| III | 80 (25) | 49 (32) | 31 (19) |  |
| Overall survival (months) | 81 (21-107) | 46 (12-103) | 83 (42-NE) | **<0.001** |
| Progression-free interval (months) | 24 (8-NE) | 13 (6-56) | 33 (13-NE) | **<0.001** |

Continuous variables are shown as mean ± standard deviation; median (interquartile range), and compared between groups using Wilcoxon test; categorical variables are shown as count (percentage [%]), and compared between groups using *χ^2^* test or Fisher’s exact test; survival data were in median (interquartile range) as calculated using the Kaplan-Meier method, and compared between groups using log-rank test. Patients with unknown survival status and/or time (n=24) and patients with metastatic disease (n=3) were excluded from analysis. NE, not estimable.

**Supplementary Table 3.** Associations of Cluster 2 versus Cluster 1 with clinicopathologic factors, assessed using multivariable-adjusted logistic regression

| Variables | OR (95% CI) | *P* | *P_trend_* |
| --- | --- | --- | --- |
| Age (as continuous) | 1.01 (0.99 to 1.03) | 0.430 |  |
| Sex |  |  |  |
| Male | 1 (reference) |  |  |
| Female | 0.75 (0.43 to 1.32) | 0.318 |  |
| Histologic grade |  |  | **0.012** |
| G1 | 2.69 (1.09 to 6.62) | **0.031** |  |
| G2 | 1 (reference) |  |  |
| G3 | 0.62 (0.34 to 1.12) | 0.112 |  |
| G4 | 0.45 (0.10 to 1.94) | 0.282 |  |
| pTNM stage |  |  | **0.047** |
| I | 1 (reference) |  |  |
| II | 0.63 (0.33 to 1.20) | 0.159 |  |
| III | 0.45 (0.23 to 0.87) | **0.017** |  |

Associations of molecular subtype (Cluster 2 versus Cluster 1) with age, sex, histologic grade, and pTNM stage were estimated using multivariable logistic regression with mutual adjustment for these factors. OR, odds ratio; CI, confidence interval; NE, not estimable.

**Supplementary Table 4.** Differentially enriched molecular pathways between the two Clusters

| **Pathways** | **logFC** | ***P* value** | **adj. *P* value** |
| --- | --- | --- | --- |
| KEGG_CELL_CYCLE | 0.416987275 | 3.76E-68 | 6.99E-66 |
| KEGG_HOMOLOGOUS_RECOMBINATION | 0.488764004 | 2.63E-59 | 2.44E-57 |
| KEGG_DNA_REPLICATION | 0.583641673 | 4.57E-56 | 2.83E-54 |
| KEGG_MISMATCH_REPAIR | 0.435728831 | 2.43E-40 | 1.13E-38 |
| KEGG_OOCYTE_MEIOSIS | 0.20362039 | 1.37E-37 | 5.11E-36 |
| KEGG_COMPLEMENT_AND_COAGULATION_CASCADES | -0.43539257 | 3.54E-32 | 1.10E-30 |
| KEGG_PROGESTERONE_MEDIATED_OOCYTE_MATURATION | 0.183435469 | 5.83E-28 | 1.55E-26 |
| KEGG_LINOLEIC_ACID_METABOLISM | -0.302304509 | 3.25E-27 | 7.56E-26 |
| KEGG_TYROSINE_METABOLISM | -0.313095389 | 6.56E-26 | 1.36E-24 |
| KEGG_FATTY_ACID_METABOLISM | -0.445198847 | 9.84E-26 | 1.83E-24 |
| KEGG_ARGININE_AND_PROLINE_METABOLISM | -0.303895623 | 3.97E-25 | 6.71E-24 |
| KEGG_DRUG_METABOLISM_CYTOCHROME_P450 | -0.369244224 | 1.52E-24 | 2.36E-23 |
| KEGG_TRYPTOPHAN_METABOLISM | -0.372207646 | 3.03E-23 | 4.34E-22 |
| KEGG_PRIMARY_BILE_ACID_BIOSYNTHESIS | -0.457993857 | 4.00E-23 | 5.31E-22 |
| KEGG_VALINE_LEUCINE_AND_ISOLEUCINE_DEGRADATION | -0.411370953 | 7.15E-22 | 8.87E-21 |
| KEGG_BASE_EXCISION_REPAIR | 0.281160419 | 1.11E-21 | 1.29E-20 |
| KEGG_RETINOL_METABOLISM | -0.356188888 | 1.89E-21 | 2.07E-20 |
| KEGG_PROPANOATE_METABOLISM | -0.376901776 | 5.92E-21 | 6.11E-20 |
| KEGG_METABOLISM_OF_XENOBIOTICS_BY_CYTOCHROME_P450 | -0.325954022 | 7.74E-21 | 7.58E-20 |
| KEGG_BETA_ALANINE_METABOLISM | -0.361424027 | 9.19E-21 | 8.55E-20 |
| KEGG_PHENYLALANINE_METABOLISM | -0.300780733 | 1.17E-20 | 1.04E-19 |
| KEGG_LIMONENE_AND_PINENE_DEGRADATION | -0.431131408 | 2.44E-20 | 2.06E-19 |
| KEGG_PEROXISOME | -0.326303958 | 3.90E-20 | 3.16E-19 |
| KEGG_ADIPOCYTOKINE_SIGNALING_PATHWAY | -0.191646694 | 9.97E-20 | 7.73E-19 |
| KEGG_PPAR_SIGNALING_PATHWAY | -0.266865296 | 1.18E-18 | 8.78E-18 |
| KEGG_CITRATE_CYCLE_TCA_CYCLE | -0.33898324 | 5.99E-18 | 4.29E-17 |
| KEGG_HISTIDINE_METABOLISM | -0.282014485 | 6.33E-18 | 4.36E-17 |
| KEGG_STEROID_HORMONE_BIOSYNTHESIS | -0.293439132 | 1.12E-17 | 7.41E-17 |
| KEGG_BUTANOATE_METABOLISM | -0.325603566 | 2.31E-17 | 1.48E-16 |
| KEGG_ALANINE_ASPARTATE_AND_GLUTAMATE_METABOLISM | -0.244976931 | 2.69E-17 | 1.67E-16 |
| KEGG_NUCLEOTIDE_EXCISION_REPAIR | 0.217218033 | 1.21E-16 | 7.26E-16 |
| KEGG_ARACHIDONIC_ACID_METABOLISM | -0.191386345 | 8.27E-16 | 4.81E-15 |
| KEGG_PROXIMAL_TUBULE_BICARBONATE_RECLAMATION | -0.193989628 | 1.44E-14 | 8.11E-14 |
| KEGG_RENIN_ANGIOTENSIN_SYSTEM | -0.22341894 | 2.84E-14 | 1.55E-13 |
| KEGG_PYRUVATE_METABOLISM | -0.24557153 | 3.94E-14 | 2.09E-13 |
| KEGG_NICOTINATE_AND_NICOTINAMIDE_METABOLISM | -0.182719854 | 5.21E-14 | 2.69E-13 |
| KEGG_GLYCOLYSIS_GLUCONEOGENESIS | -0.197907323 | 1.31E-13 | 6.48E-13 |
| KEGG_P53_SIGNALING_PATHWAY | 0.135868493 | 1.32E-13 | 6.48E-13 |
| KEGG_BLADDER_CANCER | 0.147151902 | 2.35E-13 | 1.12E-12 |
| KEGG_PORPHYRIN_AND_CHLOROPHYLL_METABOLISM | -0.25874382 | 6.47E-13 | 3.01E-12 |
| KEGG_GLYOXYLATE_AND_DICARBOXYLATE_METABOLISM | -0.254049321 | 1.14E-12 | 5.15E-12 |
| KEGG_SPLICEOSOME | 0.2235798 | 1.31E-12 | 5.81E-12 |
| KEGG_GLYCINE_SERINE_AND_THREONINE_METABOLISM | -0.29754715 | 1.63E-12 | 7.07E-12 |
| KEGG_TAURINE_AND_HYPOTAURINE_METABOLISM | -0.195632143 | 2.45E-12 | 1.04E-11 |
| KEGG_NON_HOMOLOGOUS_END_JOINING | 0.205286072 | 1.04E-11 | 4.26E-11 |
| KEGG_NITROGEN_METABOLISM | -0.176466355 | 1.06E-11 | 4.26E-11 |
| KEGG_ASCORBATE_AND_ALDARATE_METABOLISM | -0.294486384 | 1.08E-11 | 4.26E-11 |
| KEGG_STARCH_AND_SUCROSE_METABOLISM | -0.204448344 | 3.42E-11 | 1.32E-10 |
| KEGG_DRUG_METABOLISM_OTHER_ENZYMES | -0.223628357 | 5.06E-11 | 1.92E-10 |
| KEGG_FOLATE_BIOSYNTHESIS | -0.2508501 | 1.39E-10 | 5.17E-10 |
| KEGG_PATHOGENIC_ESCHERICHIA_COLI_INFECTION | 0.141907742 | 6.44E-10 | 2.35E-09 |
| KEGG_PENTOSE_AND_GLUCURONATE_INTERCONVERSIONS | -0.24194574 | 2.31E-09 | 8.27E-09 |
| KEGG_ALPHA_LINOLENIC_ACID_METABOLISM | -0.140079582 | 1.49E-08 | 5.24E-08 |
| KEGG_LYSINE_DEGRADATION | -0.178353709 | 2.37E-08 | 8.15E-08 |
| KEGG_REGULATION_OF_AUTOPHAGY | -0.115593752 | 4.74E-08 | 1.60E-07 |
| KEGG_BIOSYNTHESIS_OF_UNSATURATED_FATTY_ACIDS | -0.195830806 | 1.81E-07 | 6.01E-07 |
| KEGG_ALZHEIMERS_DISEASE | -0.140743121 | 2.12E-07 | 6.93E-07 |
| KEGG_GLYCOSAMINOGLYCAN_DEGRADATION | -0.143661864 | 2.22E-07 | 7.12E-07 |
| KEGG_OXIDATIVE_PHOSPHORYLATION | -0.214321288 | 2.33E-07 | 7.34E-07 |
| KEGG_PRION_DISEASES | -0.135204712 | 3.12E-07 | 9.68E-07 |
| KEGG_ABC_TRANSPORTERS | -0.121812244 | 9.81E-07 | 2.99E-06 |
| KEGG_GLYCOSPHINGOLIPID_BIOSYNTHESIS_GANGLIO_SERIES | -0.147776831 | 1.15E-06 | 3.42E-06 |
| KEGG_PARKINSONS_DISEASE | -0.197054014 | 1.16E-06 | 3.42E-06 |
| KEGG_RNA_DEGRADATION | 0.103867 | 7.73E-06 | 2.25E-05 |
| KEGG_GALACTOSE_METABOLISM | -0.108945963 | 1.00E-05 | 2.82E-05 |
| KEGG_GLUTATHIONE_METABOLISM | -0.115818608 | 1.68E-05 | 4.58E-05 |
| KEGG_OTHER_GLYCAN_DEGRADATION | -0.124306351 | 7.80E-05 | 0.00020161 |
| KEGG_AMINOACYL_TRNA_BIOSYNTHESIS | -0.119537342 | 0.000467982 | 0.001130451 |
| KEGG_PROTEIN_EXPORT | -0.122715858 | 0.000670531 | 0.001598959 |
| KEGG_TERPENOID_BACKBONE_BIOSYNTHESIS | -0.114894887 | 0.005706801 | 0.011794056 |
